# Distribution and pollination services of wild bees and hoverflies along an altitudinal gradient in mountain hay meadows

**DOI:** 10.1002/ece3.7924

**Published:** 2021-07-21

**Authors:** Kevin Baumann, Julia Keune, Volkmar Wolters, Frank Jauker

**Affiliations:** ^1^ Department of Animal Ecology Justus Liebig University Giessen Giessen Germany

**Keywords:** apidae, community assembly, ecosystem service, environmental filtering, flower flies, syrphidae

## Abstract

Extensively managed and flower‐rich mountain hay meadows, hotspots of Europe's biodiversity, are subject to environmental and climatic gradients linked to altitude. While the shift of pollinators from bee‐ to fly‐dominated communities with increasing elevation across vegetation zones is well established, the effect of highland altitudinal gradients on the community structure of pollinators within a specific habitat is poorly understood. We assessed wild bee and hoverfly communities, and their pollination service to three plant species common in mountain hay meadows, in eighteen extensively managed yellow oat grasslands (*Trisetum flavescens*) with an altitudinal gradient spanning approx. 300 m. Species richness and abundance of pollinators increased with elevation, but no shift between hoverflies and wild bees (mainly bumblebees) occurred. Seedset of the woodland cranesbill (*Geranium sylvaticum*) increased with hoverfly abundance, and seedset of the marsh thistle (*Cirsium palustre*) increased with wild bee abundance. Black rampion (*Phyteuma nigrum*) showed no significant response. The assignment of specific pollinator communities, and their response to altitude in highlands, to different plant species underlines the importance of wild bees and hoverflies as pollinators in extensive grassland systems.

## Graphical abstract

To date, the effects of highland altitudinal gradients, within a specific habitat type, on pollinator community structures are poorly understood. We thus evaluate community structures of wild bees and hoverflies and their consequences for pollination services throughout a highland altitude gradient in mountain hay meadows. Our results clearly support a similar response to altitude for both pollinator taxa when sampled in the same grassland habitat type.

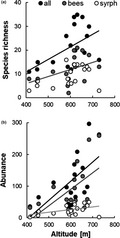

## 1 INTRODUCTION

Extensively managed grasslands, providing a significant part of the European biodiversity, are threatened by both intensification and abandonment (Cousins et al., 2015; Hilpold et al., 2018). Accordingly, a thorough understanding of community structure shifts along environmental and climatic gradients, and of the functional consequences, is required. A broad altitudinal range subjects mountain hay meadows to gradients in local temperature and humidity (Jäger & Frank, 2002) and provides an opportunity to better understand the interplay between environmental change and the complex interactions within communities (Dullau & Brade, 2010). Here, we aim to identify potential shifts among pollinator taxa and consequences for plant–pollinator interactions in yellow oat grasslands.

Wild bees and hoverflies are largely recognized as key pollinators of wild plants (Larson et al., 2001; Ollerton et al., 2011). Contribution of both taxa to pollination service, however, is highly dependent on the dominance structure within regional communities (Kleijn et al., 2015; Winfree et al., 2018). Wild bees and hoverflies often show contrasting responses in species richness and abundance to environmental factors, including elevation (Kearns, 1992). Regarding elevation, this has led to the assumption that the pollinator community structure in total shifts from bee‐dominated at lower altitudes to fly‐dominated communities at higher altitudes. The generality of this pattern, however, depends on the inclusion of high, alpine altitudes (Adedoja et al., 2018; Lefebvre et al., 2018), most likely because of confounding factors across altitudes such as changing vegetation zones and reduced tree canopy cover (McCabe et al., 2019). Still, the proportion of fly‐pollinated plants increases with alpine altitude and bee‐pollinated plants are invariably serviced by fewer bee species (Kalin Arroyo et al., 1982).

The strong shifts along broad altitudinal gradients in the structure of bee–fly assemblages are thus a result of environmental filtering (related to different habitat types), abiotic filtering (related to altered climatic conditions), and biotic interactions (competition among flower visitors), which is challenging to disentangle (Kleijn et al., 2015; Spasojevic & Suding, 2012). Accordingly, the more subtle interplay between plant and pollinator community structures along altitudinal gradients of the same habitat type is less clear (Arnold et al., 2009), but ruling out environmental changes related to different habitat types potentially relaxes the shift from bee‐dominated to fly‐dominated communities. We thus sampled wild bee and hoverfly communities in yellow oat grasslands along an altitudinal gradient typical for highlands and assessed pollination services (seedset) to three target plant species to address the following hypotheses: (i) The pollinator community of wild bees and hoverflies in mountain hay meadows responds similar to the highland altitudinal gradient and (ii) the seedset of the studied plant species responds directly to increased abundances of specific pollinator groups.

## 2 MATERIALS AND METHODS

### 2.1 Study sites and plant species

The study was conducted in the Vogelsberg region of Hesse, Germany, in the Nature Park “Hoher Vogelsberg,” a low mountain range reaching 774 m above sea level. The extension is 1,460 km² with 44% agricultural land, of which approx. half is grassland. Eighteen mountain hay meadows (yellow oat grassland communities) ranging between 3,883 m² and 25,720 m² in size were selected along an altitudinal gradient ranging from 413 to 728 m a.s.l. This gradient reflects a decrease in surface temperature of approx. 2°C, assuming a highland lapse rate of 6.5°C/km (Maurer et al., 2002). The two meteorological stations closest to our study sites, located at 265 m a.s.l. (Schotten) and 744 m a.s.l. (Hoherodskopf), put the local lapse rate over the study period at 5.0°C/km, but showed little differentiation in precipitation and daily sunshine hours (see Supporting Information File S4). We assessed flower visitation and pollination performance for three target plant species typical for the mountain hay meadows of the region (Happel & Nowak, 2000; Knapp, 1958): black rampion *Phyteuma nigrum* (present at 15 study sites), woodland cranesbill *Geranium sylvaticum* (present at 15 study sites), and marsh thistle *Cirsium palustre* (present at 14 study sites). For detailed information on the ecology of target plants, see Supporting Information File S1.

### 2.2 Flower visitation and pollination performance

Wild bees and hoverflies were sampled from the study plant species between end of May and end of July 2018 on clear, sunny days with little wind and temperatures above 10°C–12°C. Flower visitors (wild bees and hoverflies showing clear foraging activity including contact with stigmata and/or anthers) were sampled with an insect net, transferred into a glass vial with ether, and identified to species level (except for individuals of the *B*. *terrestris* complex) in the laboratory. Five sampling rounds of 15 min per site and flower species were performed at random patches throughout the study site for *G. sylvaticum* and *C. palustre*, respectively. Because flowers faded within the first two weeks, only two full sampling rounds were possible for *P. nigrum*.

For each survey round, flower cover and richness were estimated for the whole study site using digital photographs for comparison. Flower cover was visually estimated in 10% intervals, and flower richness was assigned into three categories (low, average, and high). For analyses, the average across all survey rounds was used. Because flower richness and cover were highly related, we only used flower richness in subsequent analyses. For *G. sylvaticum* and *C. palustre*, patch size within study site was estimated and the average patch size calculated per site. *P. nigrum* could not be assigned to patches. Patch size, however, was not an important factor in flower visitor or pollination performance analyses and was thus eliminated.

Pollination experiments encompassed 20 plant individuals of similar flower number and general appearance per site and per plant species. Half of these were tagged as study plants, and half were bagged with perforated polypropylene bags as control plants before flowering. Tagged (and bagged) plants were excluded from flower visitor surveys as described above to avoid damaging and disturbance. Plant growth and fruiting were surveyed every four days. When seeds were matured, tagged and bagged plants were collected and kept separately in paper bags until further processing. Number of seeds per plant was counted manually for *G. sylvaticum* and *C. palustre*. Seeds of *P. nigrum* (up to 2,500 seeds per plant) were counted using a seed counter (“Contador,” Pfeuffer, Germany). Seedset was calculated as the average number of seeds per flower by dividing number of seeds per plant by number of flowers per plant for tagged and control plants per site.

### 2.3 Statistical design

#### 2.3.1 Flower visitor analyses

The main factor of interest was altitude (ranging from 413 to 728 m, mean 593 m) as a continuous variable. We additionally included habitat area (log‐transformed continuous variable; original data ranging from 1,287 to 25,720 m², mean 10,046 m²) and local flowering plant species richness (a factor with three levels: high, intermediate, and low; hereafter “flower richness”) as possible confounding variables. Altitude and habitat area were not intercorrelated (Pearson's product–moment correlation: *t*_1,16_ = −1.57, *p* = 0.136). Flower richness was neither related to altitude (ANOVA: *F_2_*_,15_ = 1.36, *p* = 0.285) nor habitat area (ANOVA: *F*_2,15_ = 0.13, *p* = 0.878). We performed linear models for Gaussian and generalized linear models for Poisson data in R 3.2.3 (R Core Team, 2015) to explore effects of altitude, habitat area, and flower richness on species richness and abundance of all pollinators and wild bees and hoverflies separately. Pollinator data were pooled over all survey rounds and target plant species. Dependent variables were checked visually for normal distribution prior to analyses and normality and homoskedasticity of model residuals after analyses. Abundance of all pollinators and wild bee abundance showed a Poisson distribution, and a generalized linear model with quasipoisson family function was specified due to overdispersion. Species richness of hoverflies failed all model requirements, even after transformations. For a general idea of possible effects, we performed nonparametric Spearman's rank correlations with altitude and habitat area and a Kruskal–Wallis rank sum test with flower richness.

#### 2.3.2 Pollination service analyses

For each target plant species, we first subtracted the mean seedset of bagged control flowers from mean seedset of open flowers per site to avoid bias of variation in self‐pollination among localities. We visually checked for normality in adjusted seedset for all three target plant species and for normality and homoskedasticity of model residuals after analyses; no transformation was necessary. Next, we evaluated any direct pollinator effects using correlation matrices (seedset of each plant species vs. species richness and abundance of all pollinators and wild bees and hoverflies separately; Supporting Information File S4). In contrast to the prior flower visitor analyses, which pooled visitors of all target plant species, here we only used flower visitor data directly assessed from the respective plant species. Then, for each plant species, we ran two models: (i) an environmental model including elevation, habitat area, and flower richness as predictors, and (ii) a pollinator model. For this model, the pollinator variable with the highest correlation coefficient from the correlation matrix substituted significant environmental variables from the prior “flower visitor analyses” to differentiate between indirect environmental effects and direst pollinator effects.

## 3 RESULTS

A total of 2,009 individuals from 87 different species were collected: 1,556 (77%) wild bees and 453 (22%) hoverflies out of 44 (50%) wild bee and 43 (50%) hoverfly species. This represents 111.6 ± 80.0 (standard deviation) flower visitors (86.4 ± 75.1 wild bees, 25.1 ± 15.9 hoverflies) and 21.1 ± 9.0 flower visitor species (11.7 ± 5.7 wild bees, 9.4 ± 4.4 hoverflies) per site on average. By far, the most abundant wild bee genus was *Bombus* (96% of wild bee individuals in 22 species), followed by *Andrena* (2% of wild bee individuals in seven species) and *Lasioglossum* (1% of wild bee individuals in 6 species). The most abundant hoverfly genus was *Helophilus* (40% of hoverfly individuals in two species), followed by *Volucella* and *Platycheirus* (both 9% of hoverfly individuals in two respectively five species). A complete species list including environmental site parameters and coordinates is given in Supporting Information File S2.

More species were collected from *G. sylvarum* (66) than from *C. palust*re (49) and *P. nigrum* (23). While species were evenly distributed among wild bees and hoverflies for the former two, *P. nigrum* was clearly wild bee‐dominated (see Supporting Information File S2 for a graphic visualization). More individuals were collected from *C. palustre* (1,162) than from *G. sylvaticum* (649) and *P. nigrum* (198). While individuals were evenly distributed among wild bees and hoverflies for *G. sylvaticum*, the other two were clearly wild bee‐dominated (see Supporting Information File S3 for a graphic visualization).

### 3.1 Pollinator community

**FIGURE 1:**
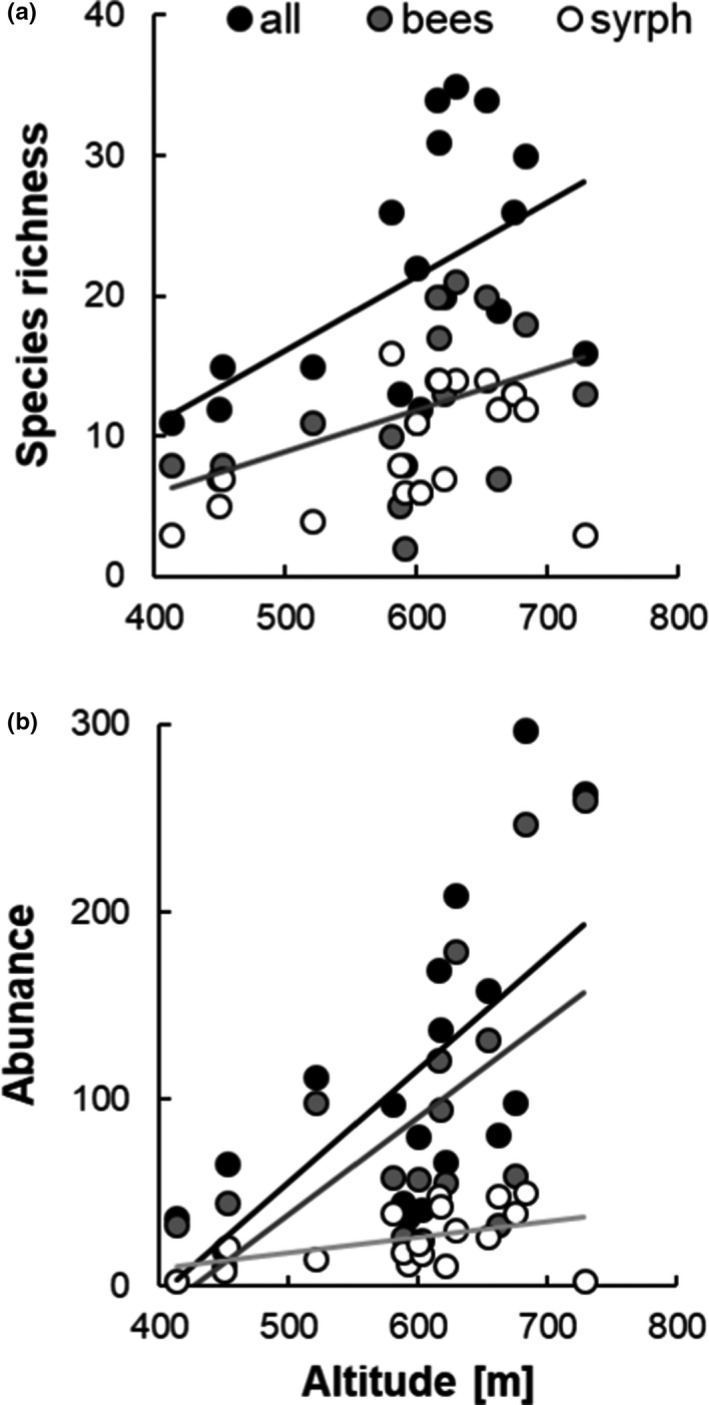
Relation between (a) wild bee, hoverfly, and combined species richness and altitude and (b) wild bee, hoverfly, and combined abundance and altitude

Altitude had a significant effect on species richness of all pollinators combined (Figure 1a, *t* = 3.56, *p* = 0.003) and on wild bee species richness (Figure 1a, *t* = 2.84, *p* = 0.014). There was no indication in the nonparametric analysis for an effect of altitude on hoverfly species richness (*S* = 629.6, *p* = 0.154, rho = 0.35). All abundance variables were significantly affected by altitude (Figure 1b, all pollinators: *t* = 3.39, *p* = 0.005; wild bees: *t* = 2.87, *p* = 0.013; and hoverflies: *t* = 2.93, *p* = 0.012). All values increased with altitude (Figure 1).

**FIGURE 2:**
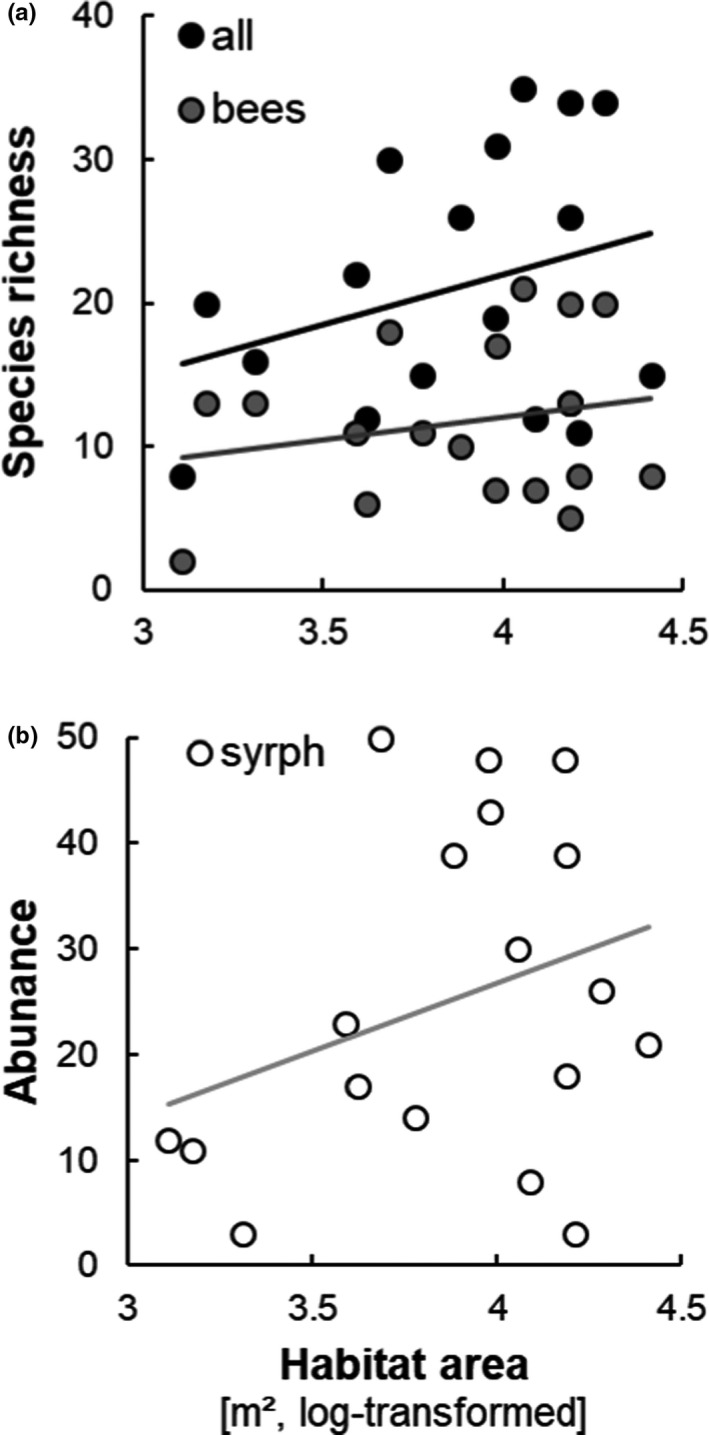
Relation between (a) wild bee, hoverfly, and combined species richness and habitat area and (b) wild bee, hoverfly, and combined abundance and habitat area

Habitat area had an additional effect on species richness of all pollinators combined (Figure 2 A, *t* = 2.84, *p* = 0.014) and hoverfly abundance (Figure 2 B, *t* = 2.63, *p* = 0.021), as well as a marginally significant effect on wild bee species richness (Figure 2a, *t* = 1.93, *p* = 0.075). There was no indication in the nonparametric analysis for an effect of habitat area on hoverfly species richness (*S* = 727.3, *p* = 0.318, rho = 0.25). Significant habitat area effects were positive throughout (Figure 2). No significant effects could be established for flower richness (full model statistics are given in Supporting Information File S4).

### 3.2 Seedset

Results are based on a total of 321,458 *P. nigrum* seeds, 5,735 *G. sylvarum* seeds, and 36,491 *C. palustre* seeds. Bagged plant individuals without access to any pollinators showed considerably lower seedset compared with open plant individuals and established a general pollination dependency, but to different degrees between target plant species. Bagged flowers of *C. palustre* developed on average 28.1 ± 17.8% of the number of seeds compared with open flowers, *G. sylvarum* 9.0 ± 7.2% and *P. nigrum* 0.9 ± 0.4% (responses of seedset in bagged and open flowers to environmental factors and pollinator variables are given in Supporting Information File S4).

No direct pollinator effect could be established for *P*. *nigrum*. Similarly, the environmental model did not yield any significant effects (Supporting Information File S4).

**FIGURE 3:**
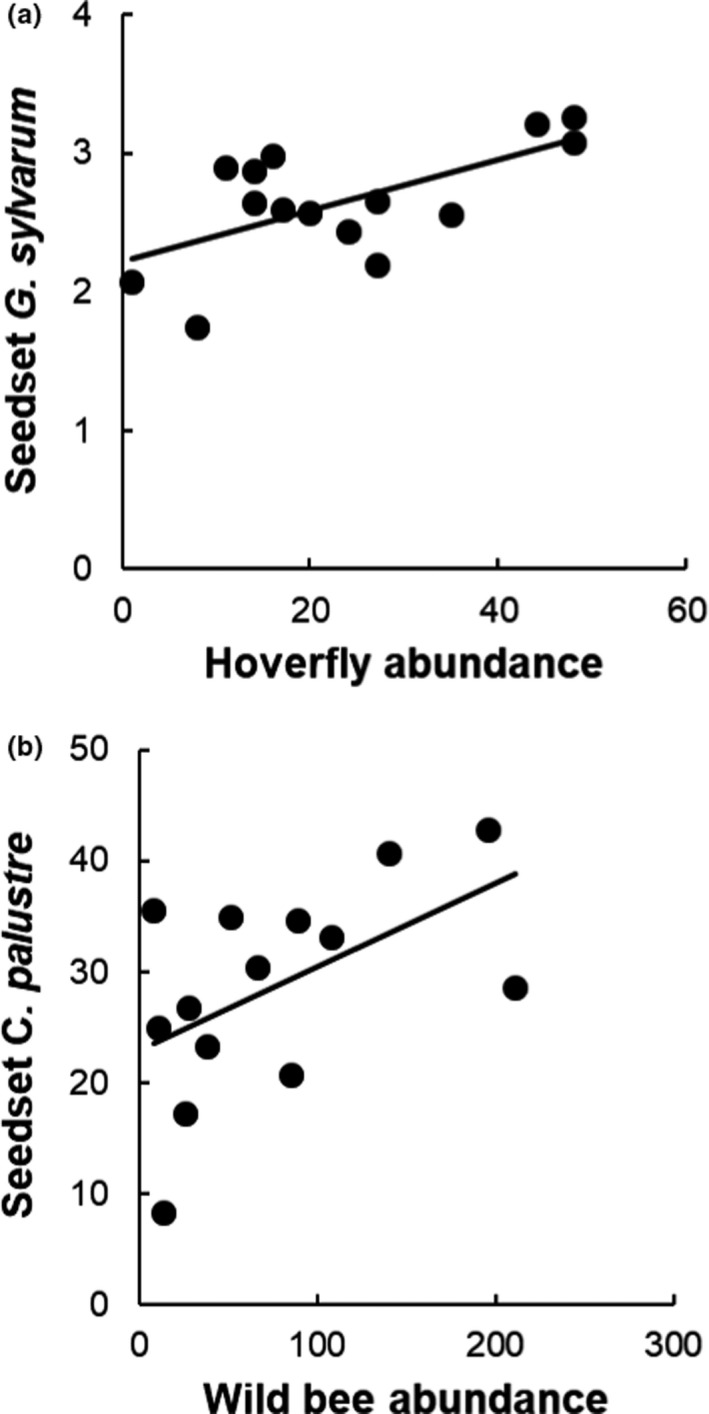
Relation between (a) seedset in *Geranium sylvaticum* and hoverfly abundance and (b) seedset in *Cirsium palustre* and wild bee abundance

For seedset in *Geranium sylvaticum*, the correlation matrix suggested direct pollinator effect of hoverfly abundance (*R* = 0.63). The environmental model did not yield any significant effects. Because hoverfly abundance was related to altitude and habitat area in the prior analysis, both were substituted in the pollinator model. Hoverfly abundance was significantly and positively related to seedset in *G. sylvaticum* (*t* = 2.50, *p* = 0.030; Figure 3a).

For seedset in *Cirsium palustre*, the correlation matrix suggested direct pollinator effect of wild bee abundance (*R* = 0.53). The environmental model did not yield any significant effects. Because abundance of wild bees was related to altitude in the prior analysis, it was substituted in the pollinator model. Wild bee abundance was positively related to seedset in *C. palustre* (*t* = 2.58, *p* = 0.030; Figure 3b).

## 4 DISCUSSION

For two out of three plant species representative of highland yellow oat grasslands, we show increased seed production when suitable pollinator taxa are abundant. These taxa, in turn, were responsive to the comparably narrow elevational gradient typical for highlands. Pollinator richness and abundance generally increased with altitude, which was attributed to wild bees rather than hoverflies. Accordingly, changes in habitat type may contribute considerably to observed shifts from bee‐dominated to fly‐dominated communities along elevational gradients covering multiple vegetation zones (McCabe et al., 2019). The wild bee community, however, was dominated by bumblebees, both in species richness and in abundance (50% of all wild bee species and 96% of all wild bee individuals). An increase in body size along altitude gradients has been shown for wild bee communities before, favoring larger species, especially bumblebees, at higher elevations (Malo & Baonza, 2002). Contrary to most other wild bees, bumblebees are less prone to unfavorable weather conditions, making them the dominant pollinator genus in highland habitats (Goulson et al., 2008; Neumayer, 1998). The suitability of the mountain hay meadows for bumblebee communities is further exemplified by a comparably high proportion of cuckoo bees (seven species out of 22, 48% of the individuals), indicating a rather intact community structure (Henson et al., 2009).

Similar to bumblebees, hoverflies often show highly diverse communities in mountainous upland regions (Devoto et al., 2005; Montoya et al., 2012). But in contrast to bumblebees, hoverflies are comparably small bodied, overheating, and dehydrating quickly (Heinrich & Pantle, 1975). At higher altitudes, conditions are thus more favorable for prolonged foraging during midday (Inouye et al., 2015; Maier & Waldbauer, 1979). Given enough nectar resources, energetic costs of endothermic regulation processes, known in some hoverfly species (Heinrich & Pantle, 1975), can be negligible, enabling activity during unfavorable conditions (Morgan & Heinrich, 1987). It is therefore surprising that species richness did not respond to altitude (although abundance did). Either the examined altitudinal range might not have been broad enough or species turnover is more important than species gain. The fact that typical species of open lowland landscapes in the region (e.g., of the genera *Episyrphus*, *Eristalis*, *Eupeodes*, *Syrphus*, and *Sphaerophoria*; see Jauker et al., 2009) were not dominant in the dataset (<20% of individuals) suggests a combination of both.

All studied target plants responded to pollinator availability with increased seedset (but to varying degree) and showed distinct pollinator communities. Although strict pollination syndromes are rare for most flowering plant species (Ollerton et al., 2009), the main visiting insect taxa were generally in line with the literature: *Phyteuma nigrum* mostly (bumble‐) bee‐visited, in terms of both species richness and abundance (Kwak et al., 1991). *Cirsium palustre* visited by equal numbers of hoverfly and (bumble‐) bee species, but more often by bumblebees (Mogford, 1974) and *Geranium sylvaticum* receiving more visits from hoverflies than bumblebees (Varga & Kytöviita, 2010). Accordingly, the respective dominant pollinator taxa showed the strongest effect on pollination success (c.f. Kleijn et al., 2015): Seedset in *C. palustre* increased with wild bee visitor abundance, and seedset in *G. sylvatucum* increased with hoverfly visitor abundance. This strong direct effect of pollinator abundance on the seedset of the target plants is not surprising (Mogford, 1974; Varga & Kytöviita, 2010), but the pollinator community analyses establish an indirect link to environmental habitat parameters linked to elevation. This indeed indicates improved pollinator availability at higher altitudes and establishes the connection between the plant community in mountain hay meadows and the availability of specific pollinator taxa. For *Phyteuma nigrum*, however, seedset was neither related to any environmental factors nor pollinator availability.

Mountain hay meadows provide important habitat for wild bees, hoverflies, and plant species. Their maintenance and improvement is an important challenge for the future. *Phyteuma nigrum* and *Geranium sylvaticum*, for example, have already experienced population declines over the past years (Bradshaw, 2009; Loton, 2014), and a general pollinator decline raises concern (Potts et al., 2010; Hallmann et al., 2017). In accordance with previous studies (e.g., Meyer et al., 2009; Steffan‐Dewenter, 2003), size of the mountain hay meadows was an important driver of species richness (mainly wild bees) and hoverfly abundance, indicating that a reduction in size alters pollinator community structure and associated pollination services (Grass et al., 2018, Jauker et al., 2019). The present study gives thus insights into promising future directions of conservation efforts for highly diverse mountainous grassland systems (Jones et al., 2018), especially since responses to altitude were similar among taxa in the bee–fly assemblages in mountain hay meadows.

## CONFLICT OF INTEREST

All authors declared no conflict of interest.

## AUTHOR CONTRIBUTIONS

**Kevin Baumann:** Data curation (equal); Formal analysis (lead); Investigation (supporting); Methodology (supporting); Supervision (supporting); Writing – original draft (lead); Writing – review and editing (equal). **Julia Christina Keune:** Formal analysis (supporting); Investigation (lead); Writing – original draft (supporting); Writing – review and editing (equal). **Volkmar Wolters:** Conceptualization (supporting); Methodology (supporting); Resources (lead); Supervision (supporting); Writing – review and editing (equal). **Frank Jauker:** Conceptualization (lead); Data curation (equal); Formal analysis (supporting); Methodology (lead); Supervision (lead); Writing – review and editing (equal).

## Supporting information

Supporting Information File S1

Supporting Information File S2

Supporting Information File S3

Supporting Information File S4

## Data Availability

All data are provided in Supporting Information File S2 and have also been submitted to Dryad https://doi.org/10.5061/dryad.8gtht76px.
