# Supplementary material for: Distribution and pollination services of wild bees and hoverflies along an altitudinal gradient in mountain hay meadows
Source: Ecol Evol. 2021 Jul 21;11(16):11345–51. doi: 10.1002/ece3.7924 (PMC8366848; doi:10.1002/ece3.7924)
Supplement: Supplementary file 1 — Supporting Information File S1 [file ECE3-11-11345-s001.docx]

**Electronic supplement to:**

*Distribution and pollination services of wild bees and hoverflies along an altitudinal gradient in mountain hay meadows*

Kevin Baumann*, Julia Keune, Volkmar Wolters, Frank Jauker

*Department of Animal Ecology, Justus Liebig University Giessen, Heinrich-Buff-Ring 24-32, D-35392 Giessen, Germany; Kevin.Baumann@allzool.bio.uni-giessen.de; +40 461 99 35716

*Supplement 1: Detailed information on target plants*

The perennial *P. nigrum* (Campanulaceae) reaches up to 50 cm in height (Schauer et al. 2012). On average 40 dark- to blackish-violet flowers of 7 - 10 mm in length are arranged in dense cylindrical inflorescences (Kwak et al. 1991; Schauer et al. 2012). The seeds are oval, reddish to brown and very small (Wheeler and Hutchings 2002). The perennial *G. sylvaticum* (Geraniaceae) reaches 20 to 60 cm in height (Asikainen and Mutikainen 2003; Schauer et al. 2012). Flowers are cup-shaped, arranged in dense inflorescences and pinkish-purple with a white base (Stroh 2014). Fruits are 17 - 21mm in size with usually five or less seeds per fruit (Asikainen and Mutikainen 2005). The perennial *C. palustre* (Asteraceae) reaches 20 to 200 cm in height. Flowers are egg-shaped to cylindric and purple colored (Schauer et al. 2012). Seeds are 2 to 3 mm in size and attached to a feathery pappus of fine cottony hairs (Schauer et al. 2012). Per plant, 300 to 2,000 seeds with an average weight of 1.5 mg are produced (van Leeuwen 1981). All three plant species are self-compatible, but pollinators increase seed production (Purschke 2006).

References

Asikainen, E. and Mutikainen, P. (2003), Female frequency and relative fitness of females and hermaphrodites in gynodioecious *Geranium sylvaticum* (Geraniaceae). Am. J. Bot., 90: 226-23

Asikainen, E. and Mutikainen, P. (2005). Pollen and resource limitation in a gynodioecious species. American journal of botany. 92. 487-94

Kwak MM, Kremer P, Boerrichter E, van den Brand C (1991) Pollination of the rare species *Phyteuma nigrum* (Campanulaceae): flight distances of bumblebees. Proc. Exper. and Appl. Entomol. 2:131–136

Purschke O (2006) Modellierung der Verbreitung von Bergwiesenarten unter Klima- und Landnutzungsszenarien in Sachsen. Diplomarbeit, Hochschule Anhalt (FH), Deutschland.

Schauer T, Caspari C, Caspari S (2012) Die Pflanzen Mitteleuropas. Über 1500 Arten. BLV Buchverlag, München.

Stroh PA (2014) *Geranium sylvaticum* L. Wood Crane’s-bill. Species Account.

van Leeuwen BH (1981) The role of pollination in the population biology of the monocarpic species *Cirsium palustre* and *Cirsium vulgare*. Oecologia 51:28–32

Wheeler BR, Hutchings MJ (2002) *Phyteuma spicatum* L. Journal of Ecology 90:581–591
