# Supplementary material for: Distribution and pollination services of wild bees and hoverflies along an altitudinal gradient in mountain hay meadows
Source: Ecol Evol. 2021 Jul 21;11(16):11345–51. doi: 10.1002/ece3.7924 (PMC8366848; doi:10.1002/ece3.7924)
Supplement: Supplementary file 2 — Supporting Information File S2 [file ECE3-11-11345-s002.docx]

**Electronic supplement to:**

*Distribution and pollination services of wild bees and hoverflies along an altitudinal gradient in mountain hay meadows*

Kevin Baumann*, Julia Keune, Volkmar Wolters, Frank Jauker

*Department of Animal Ecology, Justus Liebig University Giessen, Heinrich-Buff-Ring 24-32, D-35392 Giessen, Germany; Kevin.Baumann@allzool.bio.uni-giessen.de; +40 461 99 35716

*Supplement 2: Numbers of wild bees and hoverflies collected from the target plant species*

*
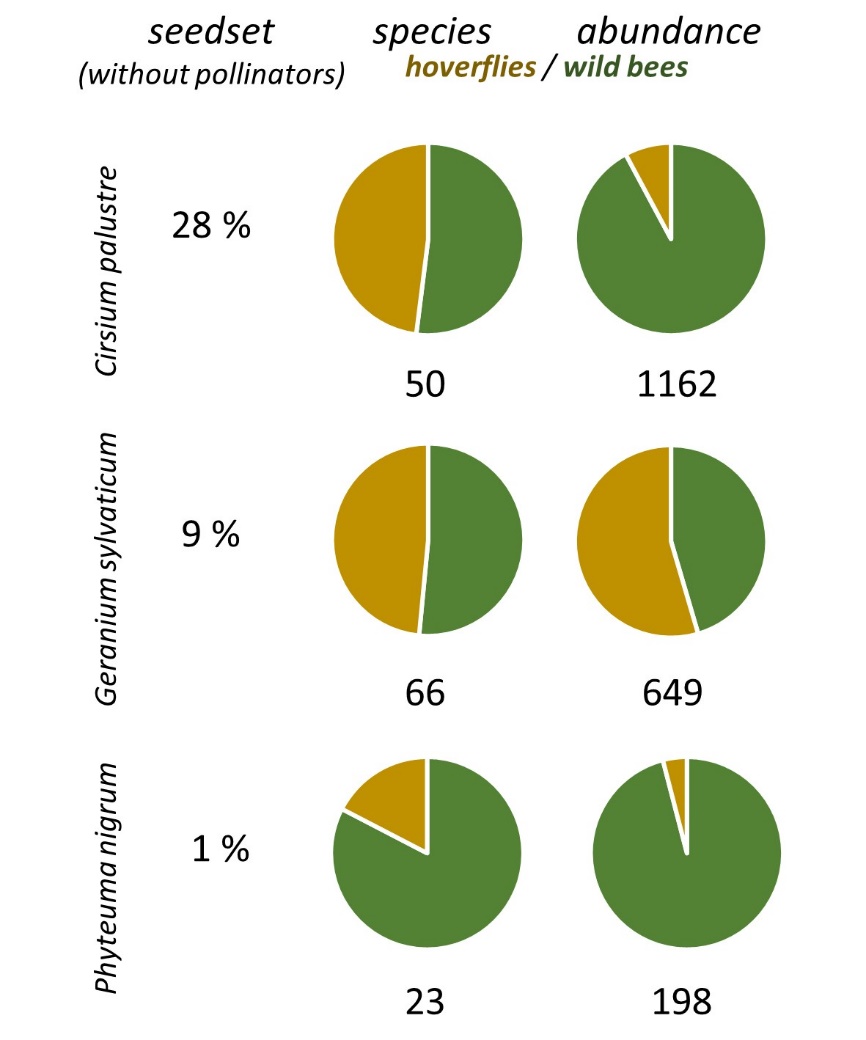
*

Fig S2: Number of wild bee and hoverfly species and individuals collected from the target plant species, as well as seedset in bagged flowers as percent of seedset in unbagged flowers.
