# Supplementary material for: Distribution and pollination services of wild bees and hoverflies along an altitudinal gradient in mountain hay meadows
Source: Ecol Evol. 2021 Jul 21;11(16):11345–51. doi: 10.1002/ece3.7924 (PMC8366848; doi:10.1002/ece3.7924)
Supplement: Supplementary file 4 — Supporting Information File S4 [file ECE3-11-11345-s003.docx]

**Electronic supplement to:**

*Distribution and pollination services of wild bees and hoverflies along an altitudinal gradient in mountain hay meadows*

Kevin Baumann*, Julia Keune, Volkmar Wolters, Frank Jauker

*Department of Animal Ecology, Justus Liebig University Giessen, Heinrich-Buff-Ring 24-32, D-35392 Giessen, Germany; Kevin.Baumann@allzool.bio.uni-giessen.de; +40 461 99 35716

*Supplement 4: supporting statistic tables*

*
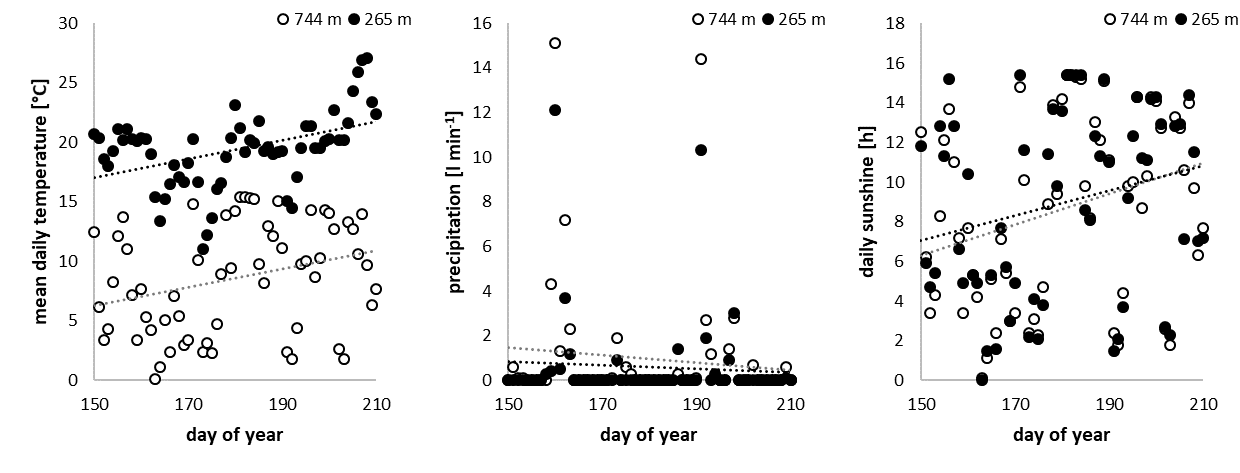
*

**Figure S4.1**: Mean daily temperature, precipitation and sunshine over the sampling period collected at the meteorological stations Schotten (265 m a.s.l.) and Hoherodskopf (744 m a.s.l.). Retrieved from www.wetterkontor.de.

**Table S4.1**: Model results for flower visitor full models. Linear Models for Gaussian distributions, Generalized Linear Models with quasipoisson family function for Poisson distributions.

|  |  | Altitude | | Area | | Flower richness | | Whole model | |
| --- | --- | --- | --- | --- | --- | --- | --- | --- | --- |
|  | Distribution | t | p | t | p | t | p | adj. R² | F_4,13_ |
| Pollinator species | Gaussian | **3.56** | **0.003** | **2.84** | **0.014** | n.s. | | 0.44 | 4.33 |
| Pollinator abundance | Poisson | **3.39** | **0.005** | 0.89 | 0.391 |  |  |  |  |
| Wild bee species | Gaussian | **2.84** | **0.014** | ***1.93*** | ***0.075*** |  |  | 0.23 | 2.30 |
| Wild bee abundance | Poisson | **2.87** | **0.013** | 0.39 | 0.708 |  |  |  |  |
| Hoverfly abundance | Gaussian | **2.93** | **0.012** | **2.63** | **0.021** |  |  | 0.36 | 3.44 |

**Table S4.2**: Correlation matrix between seedset of the three target plant species (averages over study sites; with bagged and open flowers, and open – bagged) and flower visitor data visiting the respective plant species. Gives is the correlation coefficient of pairwise Pearson correlation.

| Plant species | Seedset | Pollinator species | Pollinator abundance | Wild bee species | Wild bee abundance | Hoverfly species | Hoverfly abundance |
| --- | --- | --- | --- | --- | --- | --- | --- |
| *Phyteuma nigrum* | bagged | 0.02 | 0.06 | 0.07 | 0.07 | -0.10 | -0.18 |
|  | open | 0.26 | 0.01 | 0.39 | 0.02 | -0.14 | -0.08 |
|  | open-bagged | 0.26 | 0.01 | 0.39 | 0.02 | -0.14 | -0.08 |
|  |  |  |  |  |  |  |  |
| *Geranium sylvaticum* | bagged | -0.10 | -0.19 | -0.13 | -0.21 | -0.02 | -0.14 |
|  | open | 0.39 | 0.43 | 0.14 | 0.23 | **0.58** | **0.56** |
|  | open-bagged | 0.43 | **0.55** | 0.23 | 0.38 | **0.55** | **0.63** |
|  |  |  |  |  |  |  |  |
| *Cirsium palustre* | bagged | -0.14 | -0.36 | -0.37 | -0.37 | 0.25 | 0.32 |
|  | open | 0.26 | 0.38 | 0.31 | 0.38 | 0.08 | -0.03 |
|  | open-bagged | 0.25 | *0.52* | *0.48* | **0.53** | -0.18 | -0.31 |

**Table S4.3**: Relation between seedset in bagged flowers (average over study sites) and environmental factors.

|  |  | Spearman rho | | K-W chi² |
| --- | --- | --- | --- | --- |
| Plant species | Seedset | Altitude | Area | Flower richness |
| *Phyteuma nigrum* | bagged | 0.10 | 0.38 | 1.82 |
| *Geranium sylvaticum* | bagged | -0.11 | 0.15 | 0.19 |
| *Cirsium palustre* | bagged | -0.06 | -0.30 | *4.61* |

**Table S4.4**: Model results for environmental and pollinator full models. Linear Models for Gaussian distributions.

|  | Environmental model | | | | | |  |  |  | Pollinator model | | | | | |  |  |
| --- | --- | --- | --- | --- | --- | --- | --- | --- | --- | --- | --- | --- | --- | --- | --- | --- | --- |
|  | Altitude | | Area | | Flower richness | | Whole model | |  | Pollinator | | Area | | Flower richness | | Whole model | |
|  | t | p | t | p | t | p |  |  |  | t | p | t | p | t | p |  |  |
| *Phyteuma nigrum* | -0.80 | 0.440 | 0.53 | 0.607 |  |  | adj. R² = 0.25 | F_2,10_ = 2.19 |  | NA | | | | | | | |
|  |  |  |  |  |  |  |  |  |  | Hoverfly abundance | |  |  |  |  |  |  |
| *Geranium sylvaticum* | 1.39 | 0.195 | 0.85 | 0.413 | n.s. | | adj. R² = 0.02 | F_2,10_ = 1.06 |  | **2.50** | **0.030** | NA | | n.s. | | adj. R² = 0.30 | F_3,11_ = 3.03 |
|  |  |  |  |  |  |  |  |  |  | Wild bee abundance | |  |  |  |  |  |  |
| *Cirsium palustre* | 0.05 | 0.959 | 0.22 | 0.835 |  |  | adj. R² = -0.31 | F_2,10_ = 0.23 |  | ***2.58*** | ***0.030*** | 1.37 | 0.204 |  |  | adj. R² = 0.25 | F_4,9_ = 2.07 |
